# Response to “Reproducibility of CRISPR-Cas9 methods for generation of conditional mouse alleles: a multi-center evaluation”

**DOI:** 10.1186/s13059-021-02312-3

**Published:** 2021-04-07

**Authors:** Hui Yang, Haoyi Wang, Rudolf Jaenisch

**Affiliations:** 1 Institute of Neuroscience, State Key Laboratory of Neuroscience, Key Laboratory of Primate Neurobiology, CAS Center for Excellence in Brain Science and Intelligence Technology, Shanghai Research Center for Brain Science and Brain-Inspired Intelligence, Shanghai Institutes for Biological Sciences, Chinese Academy of Sciences, Shanghai, 200031 China; 2 State Key Laboratory of Stem Cell and Reproductive Biology, Institute of Zoology, The Chinese Academy of Sciences, Beijing, China; 3 Whitehead Institute for Biomedical Research, Cambridge, MA USA; 4 Department of Biology, Massachusetts Institute of Technology, Cambridge, MA USA

Gurumurthy et al. [1] recently reported that a method developed by Yang et al. to generate floxed allele (designated as “two donor method” by Gurumurthy et al.) [2] had poor reproducibility. They claimed that three centers could not reproduce our results on generating conditional alleles of the *Mecp2* locus and that the “two-donor method” had very low success rate on other loci.

Here, we provide our responses to these claims:

1. Our results on *Mecp2* locus published by Yang et al. have been reproduced by independent experiments in the Jaenisch (8–10% correct alleles), Yang (8% correct alleles) and Hatada’s groups (2–6% correct alleles) [3], respectively. In addition, multiple peer-reviewed publications [3–7] have successfully used this method to create conditional knockout (CKO) mice (9 out of 11 loci succeeded, 2.5% to 18% efficiency). We noticed that the efficiency of generating CKO mice by CRISPR/Cas9 could vary, which might due to different platform features or experiment conditions.
2. The conditions used by Gurumurthy et al. [1] do not correspond to the conditions used in our paper. The concentrations of CRISPR reagents used in the Gurumurthy et al.’s study [1] on the *Mecp2* locus (10 ng/μl for Cas9 mRNA, 10 ng/μl for sgRNA, and 10 ng/μl for oligos) were much lower (10-fold lower RNA and 20-fold lower oligo donor concentration) than those used in the Yang et al.’s experiments (Cas9 100 ng/μl, sgRNA 50 ng/μl and 100 ng/μl for each oligo) [2] and Yang et al.’s previous [8] and following publications [9–12]. It is well known that the concentrations of CRISPR reagents are well correlated with the genome editing efficiency.
3. We utilized piezo-driven zygote injection method in our original paper, which allows for injecting CRISPR components at much higher concentration. The difference between this method and pronuclear injection method used by Gurumurthy et al. might also contribute to the difference of successful rates.
  In general, with any genome editing method or strategy being used, the efficiencies at different genomic loci are often highly variable. In the 2013 proof of concept paper, we showed the feasibility of generating floxed allele at *Mecp2* locus using CRISPR. To assume the efficiency we demonstrated at *Mecp2* locus will be directly translated to the success rate at other genomic loci seems premature.
  We agree with the Gurumurthy et al.’s comment that the “one-donor method” offers higher success rate for generating floxed alleles in general, while the efficiency of “one-donor method” is also variable depending on the genomic loci and donor plasmid design. Before the publication of Gurumurthy et al., we also noted this, and developed a “one-donor method,” termed “Tild-CRISPR” method [12], and demonstrated the feasibility and high efficiency in generating CKO mice.
  With the fast improvement of genome editing technologies, we and many others constantly optimize our protocols. We welcome all discussions about the choice of optimal strategy for particular applications, however, we think the reproducibility of any published work can only be validated by using the exact same experimental methods and technical parameters.
